# Proceedings of Advancing the Science of Community Engaged Research (CEnR): Innovative and Effective Methods of Stakeholder Engagement in Translational Research: abstracts: Washington, DC, USA. 14-15 September 2017

**DOI:** 10.1186/s12919-019-0163-z

**Published:** 2019-04-19

## P1 Overcoming challenges of community-university research partnerships: exploring opportunities for training, support, and program development

### Deborah Hendricks, Amy Shanafelt, Sheila Riggs, Kathleen Call, Milton Eder

#### University of Minnesota CTSI, Minneapolis, MN, USA

##### **Correspondence:** Deborah Hendricks

**Background** Effective community-university research partnerships foster trust, research relevance, and application of results. Inherent challenges include competing priorities and potential burden on community partners. The University of Minnesota Clinical and Translational Science Institute Office of Community Engagement to Advance Research and Community Health funds community-university partnerships, focusing on underserved populations in research.

**Materials and methods** Progress and final reports completed by both partners ask about challenges experienced by partnerships, strategies to overcome challenges, and contributions that strengthened project outcomes. Challenges and facilitators from projects funded between 2013 and 2015 (n=21) were categorized and analyzed thematically. We also explored the dynamic between support and monitoring of funded partnerships, which facilitated identification of challenges and approaches to resolve them.

**Results** Preliminary findings include challenges in developing and applying research strategies to meet both community and university standards; community partner organizational changes, conflicting priorities (research vs. programming), complex university administrative processes, and cultural and language barriers. While recruitment was often cited as a challenge, community partners contributed to solutions, such as building rapport and providing trusted locations for research interactions. Flexibility in responding to challenges was identified by partners as key to accomplishing their goals.

**Conclusion** CTSAs funding community-university research partnerships should consider training, support and program changes that respond to common challenges. Examples include building capacity to address competing priorities and paradigms, cultivating effective communication and partnerships, streamlining institutional processes to reduce community partner burden, and formulating a roster of community organizations to be tapped if funded organizations can no longer participate fully in projects.

## P2 Design-based community building: using design to engage people and create community

### Helen Sanematsu, Sarah Wiehe

#### Indiana CTSI, Indianapolis, IN USA

##### **Correspondence:** Helen Sanematsu

Design processes can help build community and create shared understanding. An approach to design creation has helped us re-engage our membership and introduce us to non-members. Developing an infrastructure for community-engaged research can be challenging. It is difficult to create communication that is consistent while simultaneously building a network, creating programs, and serving the network being created. We wanted to create a system that would make consistent as many variables as possible, allowing us freedom to create and tailor content and continue to reach out to new audiences in ways that would make CHeP and its mission memorable and useful.

Designers and community health researchers worked together to create research methods that drew from both fields. In fact, while intended outcomes from the disciplines of design and community health are different, their processes share many similarities. In a process that spanned two years, we:

- conducted face-to-face individual and group interviews with stakeholder groups;
- surveyed members of our network online;
- hosted a two-day-long “visioning” session using “open space” methods from design research;
- analyzed our findings as a collaborative group;
- used our conclusions to fortify our Strategic Plan and as the basis for our Strategic Plan;
- wrote a new mission, vision, and values statement;
- repeated the process to develop a new name, message, visual identity, social media strategy, and overall attitude and spirit to the communication of our mission.

## P3 Evaluating the impact of stakeholder-academic resource panels

### Grisel Robles-Schrader^1^, Josefina Serrato^1^, Michael Fagen ^2^

#### ^1^Center for Community Health, Northwestern University, Evanston, IL USA; ^2^Programs in Public Health, Northwestern University, Evanston, IL USA

##### **Correspondence:** Grisel Robles-Schrader

Northwestern University launched a new program called Stakeholder-Academic Resource Panels (ShARPs) in 2015, modeled after programs at Meharry-Vanderbilt and the University of North Carolina. These custom panels bring together 8-10 community stakeholders familiar with a research topic or community of focus to offer feedback on cultural adaptations that can improve research relevance and feasibility. Researchers have a 2.5-hour session with stakeholders to review their project and request constructive feedback on a specific set of questions. At the end of the session, researchers discuss opportunities for stakeholders to continue their involvement with the study, if so desired.

This study focuses on ShARP evaluation activities aimed at measuring the sessions’ effectiveness for multiple stakeholders at multiple time points. Researchers and community stakeholders assess the functioning of each session at its end. We then follow up with researchers approximately 12 months after the session to assess longer-term outcomes and change resultant from the ShARPs. Examples of intended immediate post-session changes include greater comfort level of researchers with community-engaged research, relevant community input on the study itself, and community-oriented changes to the research design. Longer-term intended outcomes include culturally responsive adaptations that increase the relevance and feasibility of studies, new community members who understand and are engaged in research, and new partnerships between researchers and community stakeholders.

Our goal is to assess whether ShARPs provide increased opportunities for community input in research and to determine if they facilitate the development of new partnerships over time, thereby increasing community engagement in research.

## P4 Healthcare providers’ perspectives on engaging in a clinical data research network

### Kim M. Unertl^1^, Alecia M. Fair^2^, Jacquelyn S. Favours^3^, Rowena J. Dolor^4^, Duane Smoot, MD^5^, Consuelo H. Wilkins, MD, MSCI^3^

#### ^1^Department of Biomedical Informatics, Vanderbilt University School of Medicine, Nashville, TN; ^2^Vanderbilt Institute for Clinical Translational Research, Vanderbilt University, Nashville, TN; ^3^Meharry-Vanderbilt Alliance, Vanderbilt University Medical Center, Meharry Medical College, Nashville, TN; ^4^Duke Clinical Research Institute, Duke University School of Medicine, Durham, NC; ^5^Department of Medicine, Meharry Medical Center, Nashville, TN USA

##### **Correspondence:** Consuelo H. Wilkins

**Background** Partnerships between healthcare providers and researchers could increase the generalizability of research findings and increase uptake of research results across populations. However, engaging clinicians in research is challenging. The purpose of this study is to elicit healthcare provider perspectives on barriers to involvement in and attitudes towards research.

**Materials and methods** Using a multi-level community engagement approach, the Mid-South Clinical Research Data Network conducted semi-structured interviews with clinicians from various disciplines and healthcare settings. Inductive content analysis was used to analyze the data for emerging themes using Dedoose.

**Results** The 59 participants include physicians, nurses, pharmacists, and other clinicians, representing a range of healthcare settings including hospitals, private practice, and community health centers. Emerging themes were 1) desire to participate in research relevant and beneficial to a practice’s patient population, 2) need to maintain efficiency of provider/practice, 3) desire for clarity regarding roles and time commitment, 4) need for compensation, incentives, or public recognition for involvement, 5) need to maintain trust of patients, and 6) need to become more familiar with research and opportunities available to providers.

**Conclusion** This study provides an in-depth understanding of the reasons providers decide to or not to be involved in research. It also demonstrates how to improve academic-provider partnerships, as well as increase providers’ level of involvement and/or responsibilities on research projects. Findings can be used to guide the development of strategies to better engage providers in research in clinical settings, which could ultimately improve patient outcomes.

## P5 Building bridges between a community and an academic medical center via community tours

### Megan B. Irby^1^, Keena R. Moore^1^, Mary Lynn Wigodsky^2^, Twana Roebuck^2,3^, Phillip Summers^1^, Scott D. Rhodes^1^

#### ^1^Program in Community Engagement, Clinical and Translational Science Institute, Wake Forest School of Medicine, Winston-Salem, NC, USA; ^2^ Community Member, Winston Salem, NC, USA; ^3^Experiment in Self-Reliance, Inc., Winston-Salem, NC, USA

##### **Correspondence:** Keena R. Moore

To better appreciate the root causes of health inequalities, faculty, administrators, and staff of academic medical centers can benefit from understanding the social determinants of health (SDH) within their local communities. Wake Forest School of Medicine (WFSM) historically has faced challenges in reaching the vulnerable communities it serves and conducting research within those communities. The Program in Community Engagement of the Wake Forest Clinical and Translational Science Institute (CTSI), in collaboration with community partners, implements community tours to enhance knowledge of underlying causes of health disparities, improve cross-cultural understanding and communication with patients, and build awareness of community resources (assets) that can be harnessed to affect health. Over the past three years, six day-long tours have been conducted with 60 faculty, administrators, and staff representing various disciplines (e.g., neurology, geriatrics, and pediatrics). Tours include routes through under-resourced neighborhoods and visits to community resources (e.g., food banks, clinics, schools, community centers, churches, and grocery stores). Evaluations are administered to assess program quality with 100% of participants reporting enhanced understanding of access-to-care barriers and how SDH impact health; 80% acknowledged the experience would impact interactions with patients and future research study designs, and 100% agreed they would recommend the tour to colleagues. Feedback from community partners and participants guided quality improvements with each tour iteration. This work advances the science of community-engaged research by partnering with community organizations to highlight the needs, priorities, and assets of communities served by WFSM, an approach intended to ensure that care provided and research conducted are inclusive of SDH.

## P6 Engaging youth advocates in community-based participatory research: the health of youth farmworkers in North Carolina

### Andreina Malki, Taylor J. Arnold , Jackeline Leyva, and Alejandra Monjarez

#### Student Action with Farmworkers, Durham, NC USA

##### **Correspondence:** Andreina Malki

This study outlines one of the central components of community engagement in an ongoing Community-Based Participatory Research (CBPR) study examining the health and safety of hired Latino youth farmworkers in North Carolina. This mixed-methods study builds on a 20-year partnership between Wake Forest School of Medicine investigators and a farmworker advocacy organization, Student Action with Farmworkers (SAF). The CBPR design incorporates youth from SAF’s *Levante* Leadership Institute, which works with North Carolina farmworker youth to build leadership skills and prepare them for higher education in all aspects of the research process, including the development of the grant application. Two *Levante* youth are paid co-investigators, serving in leadership roles as liaisons between academic investigators and the *Levante* youth. Applying concepts, they learned through the program, *Levante* co-investigators have worked with academic investigators to use a popular education framework to carry out educational sessions, elicit feedback on survey instruments, and make connections between theory and practice with the *Levante* youth. In subsequent years, *Levante* youth will use theater and arts to disseminate research study results to their communities. This project demonstrates the possibilities for enhancing community participation, specifically that of youth, to strengthen scientific studies while building local capacity to foster positive social change.

## P7 A community-academic partnership to understand the correlates of successful aging in place

### Kimberly S. Vasquez^1^, Dozene Guishard^2^, William Dionne^2^, Caroline Jiang^1^, Cameron Coffran^1^, Andrea Ronning^1^, Glenis George-Alexander^1^, Barry S. Coller^1^, Jonathan N. Tobin^1,3^, Rhonda G. Kost^1^

#### ^1^Center for Clinical and Translational Science, The Rockefeller University, New York, NY (RU-CCTS), USA; ^2^Carter Burden Network, New York, NY (CBN), USA; ^3^Clinical Directors Network New York, NY (CDN), USA

##### **Correspondence:** Rhonda G. Kost

**Background** The Rockefeller University CCTS, Clinical Directors Network, and Carter Burden Network (a multi- site senior services organization serving East Harlem, NY) formed a community-academic partnership to develop a simple validated surrogate measure of overall health status in this population. Many CBN seniors are racial/ethnic minorities, low-income, and suffer chronic conditions, depression, and food insecurity. Multiple biological, musculoskeletal, psychosocial, and nutritional factors contribute to frailty, which has been defined variously in senior health outcomes research. The CTSA-funded pilot project aims to 1) engage CBN seniors (n=240) and stakeholders in priority-setting, joint protocol-writing, and research conduct, analysis, and dissemination, 2) characterize the health status of the CBN seniors using validated measures, and 3) establish database infrastructure for current and future research.

**Materials and methods** CEnR-Navigation was used for partnership development and to engage seniors/stakeholders to refine priorities and research design, provide feedback on conduct, and analyze and disseminate results. Standard physical measurements and validated survey instruments were used to collect multiple assessments. The primary outcome is frailty as measured by validated walk/balance tests. Secondary outcomes include measures of engagement and association of services/activities participation with the primary outcome.

**Results** 29 CBN-tenants joined three engagement sessions to align study design with client priorities. Two CBN directors served as site PI and co-investigator on the study. Assessments continued through Fall 2017.

**Conclusion** A simple validated frailty measure in seniors will enable community-academic partners to accelerate community-based translational research addressing senior health.

## P8 Partnering with teachers to enhance Henrietta Lacks High School Symposium

### Crystal C. Evans^1^, Nancy Kass^1^, Daniel Ford^1^, David Lacks^2^, Jim Potter^1^, Barbara Bates Hopkins^1^, Delana Penn^3^, Darcenia McDowell^1^, Cheryl Dennison Himmelfarb^1^, Christine Weston^1^

#### ^1^Johns Hopkins University Institute for Clinical and Translational Research Community Engagement Program, Baltimore, MD, USA; ^2^Community member, Grandson, Henrietta Lacks, Baltimore, MD, USA; ^3^Baltimore City School System, Baltimore, MD, USA

##### **Correspondence:** Crystal C. Evans

**Background** After the publication of *The Immortal Life of Henrietta Lacks* in 2009, the Johns Hopkins School of Medicine decided to promote discussion about Henrietta Lacks, including her role in medical discovery and ethics with the community, especially with young people in Baltimore. The Community Engagement Program (CE) of the Johns Hopkins Institute for Clinical and Translational Research works closely with the family of Henrietta Lacks, the Community Research Advisory Council, and partners to conduct the Henrietta Lacks High School Symposium with the financial support of the Johns Hopkins School of Medicine and Hospital, a $40,000 scholarship for a local graduating high school student. Following student and teacher request, the symposium has involved a student-centered focus.

**Materials and methods** High school teachers, basic scientists, bioethicists, and C-RAC members developed a program that included 1) a 90-minute lecture on Henrietta Lacks and 2) 2-hour science, ethics/social justice, art, or community engagement labs, with presenters from the Lacks family, Johns Hopkins, and high school students. The program was promoted to 17 schools through in-person visits, phone calls, and e-mail.

**Results** 249 students registered from 12 schools. 181 students and 17 teachers attended. 58% of the students participated in the science: 25% Ethics/Social Justice, 11% Community Engagement, and 6% Art labs. 97 students and 12 teachers completed the evaluation survey.

**Conclusion** 91% of the teachers rated the program as very interesting, engaging, and student-centered. 71% of students reported a better understanding of the Henrietta Lacks story. 68% reported increased interest in scientific research careers, and teachers and students offered to help plan future programs.

## P9 Community Day at the IRB: de-mystifying the IRB and enhancing community understanding of human subjects protection in research – or – what happens behind the IRB curtain?

### Frederick Luthardt^1^, Crystal Evans^1^, Barbara Bates-Hopkins^2^, Janet Johnson^3^, Reverend Calvin Keen^4^

#### ^1^Johns Hopkins University School of Medicine, Baltimore, MD USA; ^2^Johns Hopkins University Bloomberg School of Public Health, Baltimore, MD, USA; ^3^Johns Hopkins Community Research Advisory Council, Baltimore, MD, USA; ^4^Memorial Baptist Church of Baltimore City, Baltimore, MD, USA

##### **Correspondence:** Frederick Luthardt

**Background** The existence and activities of Institutional Review Boards (IRBs) are largely mysterious to people in many communities. This problem is exacerbated by a pervasive distrust among many people concerning human subjects research (HSR). As potential participants are drawn from these same populations, we hypothesize that when IRB activities are de-mystified, community members will feel more confident that as research participants, they are truly valued as stakeholders, partners, and persons.

**Materials and methods** To foster this shift in perspective, a “Community Day at the IRB” program was initiated at Johns Hopkins University (JHU) in Baltimore, Maryland, where members of the East Baltimore community and community advisory boards attended a half-day session with the JHU IRB. The program includes a meeting with IRB staff, an introduction to IRB operations, and attending an IRB meeting. The session attendees then meet with the IRB's community representative for a question and answer period. Using a post-meeting survey, the attendees rated their experience, gauged the knowledge attained, and offered their comments and suggestions about how community engagement could be improved.

**Results** Three sessions have been completed, with a total of 12 attendees completing the surveys. All participants had a favorable response to the event, with a consensus agreeing that community representation could be augmented.

**Conclusion** This pilot project demonstrates that public understanding of research and research oversight is necessary to generate trust. Providing evidence that IRBs, researchers, and communities can operate from a position of mutual respect enhances the likelihood of research findings benefitting all communities.

## P10 Student-led community engagement and service learning projects in underserved communities

### Ruby Thomas, Beverly Taylor, Desiree Rivers, Carla Durham-Walker

#### Morehouse School of Medicine, Department of Community Health and Preventive Medicine, Atlanta, GA USA

##### **Correspondence:** Ruby Thomas

For more than two decades, Morehouse School of Medicine has trained its first-year medical students in community engagement and service learning via a year-long Community Health course. During this course, students participate in community engagement activities, conduct a community assessment, and design, implement, and evaluate an intervention to address the community’s needs. The purpose of the course is to provide opportunities for students to serve in underserved communities while learning basic skills in public health, community assessment, and community evaluation [1]. Communities of elementary school-aged children, elderly, and homeless women and children in an urban setting are engaged in activities including tutoring and mentoring, computer classes for job seeking and interviewing skills, and health and nutrition workshops. Service learning projects have resulted in both short-term and long-term gains for the community. Short-term results include increased confidence in computer and resume skills and increased knowledge in stress management. Long term, students have been able to address longstanding community needs, such as obtaining lockers for homeless shelter residents to improve privacy, funding bus passes for transportation to/from jobs, and getting a pedestrian beacon at a senior housing complex where traffic injury risk is high. Student-led service learning projects can help address specific community needs while teaching community engagement. With longstanding relationships, these projects can create sustainable improvements in the community and community organizations.

**Reference**

1. McNeal M, Blumenthal D. Innovative ways of integrating public health into the medical school curriculum. Am J Prev Med. 2011: 41(4S3):S309-S311.

## P11 Inclusion and integration of community and patient perspectives in review of pilot grant applications

### Patricia Piechowski^1^, Adam Paberzs^1^, Elizabeth LaPensee^2^

#### ^1^Community Engagement Program, Michigan Institute for Clinical & Health Research, University of Michigan, Ann Arbor, MI, USA; ^2^Pilot Grant Program and Research Development Core, Michigan Institute for Clinical & Health Research (MICHR), Ann Arbor, MI, USA

##### **Correspondence:** Patricia Piechowski

The recent funding opportunity announcement for the Clinical and Translational Science Award (CTSA) emphasizes the importance of engaging patients and communities as active partners in the full spectrum of translational research. This is a challenge in earlier phases of translational research, such as pre-clinical and clinical studies (i.e. T1-T2). Since 2008, the Michigan Institute for Clinical and Health Research (MICHR), a CTSA-funded institution, has engaged community partners in the review of pilot grant applications. However, involvement of community partners was often limited to later phase translational research. Recently, the MICHR Community Engagement and Pilot Grant Programs developed new strategies to 1) integrate community and patient perspectives in review of earlier phase translational research, 2) enhance training and education for community and patient reviewers, and 3) evaluate specific aspects of the review process. Community and patient partners now serve as members of MICHR's Scientific Review Committee (SRC) and review T1-T4 translational research alongside scientific reviewers. To educate about the review process and evaluation of applications, reviewers receive a specialized blended learning experience via webinar training, mock reviewing of real pilot grant applications, and observing a study section in action. A post-review survey and phone debrief is used to assess aspects of their participation (e.g., understanding review criteria, perceived group dynamics at study section, etc.) and areas for training improvement. This study describes the strategies, evaluation findings, and lessons learned from recent funding rounds, as well as future efforts to involve patients and communities in research activities that span the translational spectrum. The project described was supported by the National Center for Advancing Translational Sciences, Grant UL1TR000433.

## P12 Achieving health equity through health department-academic partnerships and community-engaged public health research: Healthy Chicago 2.0

### Jen Brown^1^, Lisa Masinter^2^, Nik Prachand^2^, Anne Posner^2^, Sarah Rittner^3^, Marc Atkins^4^, Pankaja Desai^5^, Doriane Miller^6^

#### ^1^Northwestern University, (ARCC), Chicago, IL, USA; ^2^ Chicago Department of Public Health, Chicago, IL, USA; ^3^ AllianceChicago, Chicago, IL, USA; ^4^ University of Illinois-Chicago, Chicago, IL, USA; ^5^ Rush University, Chicago, IL, USA; ^6^University of Chicago, Chicago, IL, USA

##### **Correspondence:** Jen Brown

In 2016, the Chicago Department of Public Health (CDPH) released Healthy Chicago 2.0 (HC2.0), a four-year strategic plan for the city’s public health system. The plan, developed collaboratively with diverse community organizations and city residents, places an emphasis on achieving health equity by focusing on root causes of health issues. The plan identifies 10 priority areas including reducing violence, economic development, improving educational opportunity, and data and research. Related to the research priority, HC2.0 and CDPH have been working closely with the Chicago Consortium for Community Engagement (C3). Established in 2009, C3 is a network of academic research institutions and community stakeholders that connects and leverages the resources of the community engagement programs of Chicago’s three Clinical and Translational Science Institutes (CTSIs): Northwestern University, University of Chicago/Rush University, and University of Illinois-Chicago. In 2015, CDPH joined the C3 Executive Committee and, together with C3 academic members and other community stakeholders, have been working to better align the resources of the CTSIs with HC2.0 priorities. Objectives include the development of a citywide research agenda framed around HC2.0 priorities, the establishment of an Office of Research and Evaluation at CDPH (headed by a new CDPH Director position to be collaboratively funded by the Chicago CTSIs), and initiatives to support the local dissemination of research findings directly to non-academic community audiences. We hope this systematic approach can serve as a model to develop other community-academic partnerships that include health departments and public health stakeholders to promote a full translational research agenda for CTSI programs nationally.

## P13 Health outcomes that matter: Engaging patients, community, and health system stakeholders to establish PCOR priorities

### Pamela Maxson^1^, Nadine Barrett^1^, Jennifer Gierisch^1^, Ebony Boulware^2^, Michelle Lyn^2^

#### ^1^Duke CTSI Community Engagement, Durham, NC, USA; ^2^Duke CTSI, Durham, NC, USA

##### **Correspondence:** Pamela Maxson

Durham and the Duke Center for Community and Population Health Improvement have launched a concerted effort to co-develop an agenda for patient-centered outcomes research (PCOR) on outcomes that matter to our local community. The primary goal is to convene a diverse membership of patients, healthcare providers, researchers, and community stakeholders to establish a 5-year PCOR roadmap to improve patient-centered outcomes in Durham.

This two-year effort comprises a series of community meetings, culminating in a patient-centered community-engaged consortium that leverages patient, community, and academic/health system strengths to inform research priority-setting activities. We aim to 1) establish a shared understanding of the need for engaging multiple stakeholders, 2) provide a discussion forum for community experiences relevant to PCOR, and 3) identify and ignite PCOR priorities relevant to patients and the Durham community.

Over 200 patients, researchers, providers, and community members attended three community meetings, from which multiple themes emerged, including 1) the need to increase trust and transparency, 2) incorporate community voices throughout the process, 3) foster equitable relationships among researchers, healthcare providers, and community, 4) increase involvement of underrepresented groups, and 5) improve dissemination of findings. Top health priorities identified for community-engaged research include education, mental health, obesity, access to affordable housing, diabetes, heart disease, and cancer.

Building on this effort, we are collaborating on community- and patient-focused research on relevant health outcomes. Leveraging the great strengths of local community, health system, and researcher stakeholders, co-developing a research agenda for patient-centered health priorities could have a profound and sustained impact on partnerships around health locally and beyond.

## P14 Mobile screenings: opening doors to improving cardiovascular health in high-risk communities

### Valerie Morales Mitchell, Marlene Peters- Lawrence, Tiffany Powell-Wiley

#### National Heart, Lung, and Blood Institute, National Institutes of Health, Bethesda, MD, USA

##### **Correspondence:** Valerie Morales Mitchell

**Learning Objectives**

1. Discuss how community partnerships with leaders of faith-based organizations, health advocates, community members, and academic leaders from area universities identified an intervention strategy promoting cardiovascular health that is compatible with the culture and life circumstances of the target community in Washington, DC.
2. Describe how community partnerships led to the development of a health and needs assessment to identify specific tools that may be utilized in a community-based health behavior change intervention targeting cardiovascular health in high-risk communities in Washington, DC.
3. Explain how mobile cardiovascular screening services in high-risk communities were designed using clearly defined processes so members of the community gain awareness of their cardiovascular health.

Our target audience consisted of community health workers and public health agencies with interest in cardiovascular disease, and the primary geographic focus of the program was urban communities with high-risk cardiovascular disease and limited access to healthcare. Poor cardiovascular health disproportionately affects populations with limited clinical care access. Interventions targeting cardiovascular health can be developed for high-risk populations using community-based participatory research (CBPR). Community partnerships can be beneficial in creating a tailored method of engaging community members from high-risk neighborhoods to improve cardiovascular health. A CBPR partnership between our National Institutes of Health research group and organizations representing Washington, DC, communities with the highest obesity rates and where physical activity (PA) and healthy nutrition resources are most limited (Wards 5, 7, and 8), developed a community advisory board in 2012. This advisory board, *DC Cardiovascular Health and Obesity Collaborative (DC CHOC)*, includes faith-based organizations and community leaders from healthcare, non-profit organizations, higher education, and local government. DC CHOC meets quarterly providing feedback on design, recruitment, and implementation of a health and needs assessment. To determine cardiovascular health factors, assess bio-psychosocial/environmental barriers to behavior change, and test tools for promoting PA and nutrition in the community, DC CHOC recommended conducting mobile screenings at Ward 5, 7, and 8 churches. Approximately 100 participants enrolled at four churches from September 2014-February 2015 (NCT: NCT01927783). We developed partnerships that helped design screenings for efficient participant engagement, identify screening volunteers, and involve community members as point-persons for further recruitment. After reviewing DC CHOC’s feedback on preliminary assessment findings, proposed targets for a behavioral health intervention were identified. Thus, community partnerships led to “mobile screenings,” a successful first step in developing a cardiovascular health intervention in high-risk Washington, DC, communities.

## P15 Progress in stakeholder-engaged research forum: best practices and key lessons learned

### Hae-Ra Han, Ashley Xu, Mona Bahouth, Kyra Waligora, Safiyyah Okoye, Joycelyn Cudjoe, Melanie Reese, Crystal Evans, Lee Bone, Cheryl Dennison-Himmelfarb

#### Johns Hopkins University, Baltimore, MD, USA

##### **Correspondence:** Hae-Ra Han

**Background** Stakeholder engagement (SE) is recognized as a fundamental process to make research more relevant, translatable, and sustainable. The Community Engagement Program of Johns Hopkins ICTR hosted the *Progress in Stakeholder Engaged Research Forum* to engage researchers and stakeholders in a group discussion to reflect on their past and current practices in planning, implementing, and evaluating a variety of aspects of SE research*.*

**Materials and methods** A total of 50 researchers and their partners of successfully funded SE research participated in five concurrent discussion sessions. Each group was charged with one of the following topics: 1) preparation of stakeholders; 2) stakeholder roles and responsibilities; 3) scope of SE; 4) process and impact evaluation of SE; and 5) approaches to promoting SE. Each group discussion was audio recorded and transcribed verbatim.

**Results** Content analysis resulted in 18 themes. Examples of main themes included: mission driven versus “checking the box,” “mayor of the block,” community promotion and advancement, mentorship, being a participant, being engaged in all stages of the research process, tension between clinical group of interest and researchers, lack of dissemination of research findings to participants, needing both quantity and quality measures (not either/or), considering “culture of goal,” being cautious about getting too formulaic about the quality and applying “Leapfrog,” community engagement from the beginning (iterative process), and transparency.

**Conclusion** The findings highlight the need for enhanced SE training and opportunities for both stakeholders and researchers. We plan to disseminate best practices for SE research while making strategic connections between stakeholders and researchers.​

## P16 Exploring African-American Baby Boomers’ perceptions of electronic health records: a case study

### Alesha Ray

#### Northcentral University, Prescott, AZ, USA

Information exchange is a vital platform in healthcare. The transformation of healthcare resulting from the implementation of electronic health records (EHRs) will affect not only providers, but also patients. However, viewing health records digitally has caused some concern among the African-American patient population. This study addressed the perceptions of African-American Baby Boomer patients about EHRs (digital health management) and what factors (inequalities, if any) impacted their views. Using critical race theory (CRT) as a theoretical foundation, this qualitative research explored their perceptions of EHRs. The goals of the study were to 1) determine African-American Baby Boomers’ knowledge and use of information technology (IT), 2) record their perceptions of EHR and what factors (inequalities, if any) impacted their views of digital management, and 3) determine whether the participants lacked trust in the healthcare system. A focus group was conducted with nine African-American participants between the ages of 50 and 68 years. CRT was used to explore the rudiments of business and public administration by addressing dynamics of a specific group of people impacted by the public issue of EHRs. The findings provided understanding to the field of business and public administration so that government leaders and officials will be able to help resolve the challenges that this population faces and how this group will not be omitted from this change. In addition, future qualitative studies could examine socioeconomic (SES) factors as a variable. Using this criterion could impact results, as literature has suggested that SES is a contributor to differences in healthcare [1]. Application of this study and subsequent research could help African-American Baby Boomers develop confidence in technology advances of healthcare.

**Reference**

1. Shi L, Stevens GD. Community Determinants and Mechanisms of Vulnerability. In: Shi L, and Stevens GD, editors. Vulnerable populations in the United States. 2^nd^ edition. Jossey-Bass; 2010.

## P17 Measures of trust and willingness to participate in research

### Victoria Villalta-Gil^1^, Jennifer Cunningham- Erves^2^, Alecia M. Fair^3^, Jacquelyn L. Favours^1^, Rowena J.Dolor^2,3,4^, Duane Smoot^2^, Consuelo H. Wilkins^1^

#### ^1^Meharry-Vanderbilt Alliance, Vanderbilt University Medical Center, Meharry Medical College, Nashville, TN, USA; ^2^Department of Internal Medicine, Meharry Medical College, Nashville, TN; ^3^ Vanderbilt Institute for Clinical Translational Research, Vanderbilt University, Nashville, TN; ^4^Duke Clinical Research Institute, Duke University School of Medicine, Durham, NC

##### **Correspondence:** Victoria Villalta-Gil

**Background** Measures of trust in research exist, but no comparison between trust scales has yet been conducted. We aim to compare the validity and reliability of two trust scales and examine the relationship between trust and willingness to participate in research.

**Materials and methods** Adults (ages 18+ years) were randomly assigned to complete one of two surveys, which were identical other than including one of two scales assessing trust in medical research [1-2]. The surveys also assessed willingness to participate in research and barriers to participation. We computed Cronbach’s alpha to determine the internal consistency reliability of the trust scales and conducted linear regression models to assess predictors of willingness to participate.

**Results** Cronbach’s alphas for both trust scales were >0.8. Of 2,722 respondents, 1,335 participants were included in model A, Mainous trust scale (R=0.340, F=34.66***), and 1,387 in model B, Hall trust scale (R=0.282, F=29.81***). In both models, trust was the main predictor of willingness to participate (Mainousβ=-0.241; Hallβ=-0.220). In model A, age, income, and health numeracy and literacy were significant predictors. In model B, health numeracy and race were significant predictors. Post-hoc correlation analysis showed differential associations between each trust scale and the remaining variables.

**Conclusion** Trust, regardless of measure, remains as the main predictor of willingness to participate in research. However, the scales differ in affecting the weight that other variables, such as race, have in characterizing factors that predict willingness to participate in research.

**Reference**

1. Hall MA, Camacho F, Lawlor JS, DePuy V, Sugarman J, Weinfurt K. Measuring trust in medical researchers. Med Care. 2006; 44(11):1048-1053.

2. Mainous AG, Smith DW, Geesey ME, Tilley BC. Development of a measure to assess patient trust in medical researchers. Ann Fam Med. 2006; 4(3):247-252.

## P18 Community engagement through coalition building to enhance public transportation and promote health

### Phillip Summers^1^, Elim Chao^1^, Paula McCoy^2^, Mark Kirstner^3^, James Perry^4^, Scott Rhodes^1^

#### ^1^Wake Forest School of Medicine, Winston-Salem, NC, USA; ^2^Neighbors for Better Neighbors, Winston-Salem, NC, USA; ^3^Piedmont Authority for Regional Transportation, Greensboro NC; ^4^Winston-Salem Urban League, Winston-Salem, NC, USA

##### **Correspondence:** Phillip Summers

Increased access to public transportation has been identified by the Centers for Disease Control and Prevention as a high-impact policy for improved public health. Community coalitions have been an effective strategy to promote policy change. The Stakeholder Advisory Committee (SAC) of the Wake Forest Clinical and Translational Science Institute (CTSI) (comprised of more than 40 lay community members and representatives from community-based organizations and Wake Forest School of Medicine (WFSM) in Winston-Salem, NC) advises the CTSI on community needs, priorities, and assets and assists in translating research into improved public health. The SAC prioritized public transportation as a critical policy issue and established the Transportation Coalition. The Coalition has convened monthly for the past year. Coalition meeting attendance averages 18 people per meeting, and attendees represent broad constituencies, including Winston-Salem residents, community-based organizations, local universities, the local transportation authority, local government, and WFSM. Coalition members identified short-term policy objectives and strategized action steps. For example, by participating in City Council meetings, Coalition members presented local and national data linking access to transportation with economic and health outcomes and advocated for extended bus services into local communities that had been previously isolated. The Coalition also helped the local transportation authority to craft recommendations submitted to City Council that included greater service during nights and weekends. Although short-term successes have been made, the Coalition has also developed long-term policy objectives and is developing action steps to further promote community health through increased access to public transportation.

## P19 Researcher perspectives on embedding community stakeholders in T1-T2 research: a potential new model for full-spectrum translational research

### Sheba George^1^, Rachelle Bross^2^, D’Ann Morris^3^, Norma Mtume^4^, Keith Norris^3^, Ibrahima Sankare^3^, Teresa Seeman^3^, Stefanie Vassar^3^, Pluscedia Williams^5^, Anna Lucas-Wright^1^, Sonya Young Aadam^6^, Arleen Brown^3^

#### ^1^UCLA/Charles Drew University, Los Angeles, CA, USA; ^2^Harbor-UCLA Medical Center/LA BioMed, Los Angeles, CA, USA; ^3^University of California, Los Angeles, CA, USA; ^4^Community Member, Oakland, CA, USA; ^5^Charles Drew University, Los Angeles, CA, USA; ^6^California Black Women’s Health Project, Inglewood, CA, USA

##### **Correspondence:** Stefanie Vassar

**Background** Effective community engagement in T3-T4 research is widespread; however, similar stakeholder involvement is missing in T1-T2 research. Recent IOM recommendations for CTSAs include having “active and substantive community stakeholder participation in priority setting and decision-making across all phases of research (T1-T4).” As part of an effort to implement a pilot program to embed community stakeholders in T1-T2 research projects, a UCLA CTSI team (co-led by community stakeholders) conducted discussion groups with researchers to assess their perspectives on this potentially innovative and synergistic opportunity.

**Materials and methods** We conducted five discussion groups with 19 basic researchers (focused on T1 or T2 research) representing four research institutions. Topics included 1) barriers/challenges to including community stakeholders in basic science, 2) skills/training required for stakeholders and researchers, and 3) potential benefits of these activities.

**Results** 1) Barriers identified included a) high levels of technicality/jargon in research settings, b) finding community stakeholders with motivation/time for participation, and c) challenges of relationship-building to establish trust and open communication. 2) Skills/training needed for community stakeholders included basic understanding of science and lab-specific knowledge, whereas researchers needed skills to communicate research concepts/relevance in lay language. 3) Participation benefits for researchers included addressing needs of surrounding (“real-life”) communities and parlaying enhanced ability to explain research in lay language to policy makers and funders.

**Conclusion** Engaging community stakeholders in basic science research proved to be challenging but with exciting potential to incorporate “real-life” community health priorities into basic research, resulting in a new model for full-spectrum translational research.

## P20 The Los Angeles County Health Profile

### Stefanie Vassar^1^, David Zingmond^1^, E. Richard Brown^1^, Gerald Kominski^1^, Rachel Louie^1^, Ying-Ying Meng^1^, Melissa Pickett^1^, Punam Parikh^1^, Ami M. Shah^2^, Sitaram Vangala^1^, Steve Wallace^1^, Arleen Brown^1^

#### ^1^University of California, Los Angeles, CA, USA; ^2^Los Angeles County Department of Health Services, Los Angeles, CA, USA

##### **Correspondence:** Stefanie Vassar

Detailed local health data can benefit communities and inspire investigators to strive collectively towards improved health. The UCLA-CTSI developed the Los Angeles County (LAC) Health Profile, a baseline report describing key indicators of health and healthcare shaped by community input to identify “hot spots” of poor health and health disparities in LAC.

Informal interviews with stakeholders from health clinics and systems, community-based organizations, academic-community partnerships, and the LAC health department were conducted to identify measures that would serve as evidence to shape community plans, health initiatives, local health policies, and program evaluation. Feedback helped to inform final indicators in six clinical domains: diabetes/obesity, cardiovascular/cerebrovascular disease, cancer, addiction, mental health, and HIV.

Using the California Health Interview Survey and hospital discharge and emergency department encounter data from the Office of State Health Planning and Development, we created profiles for the six domains mapped to LAC’s 26 health districts comprised of eight Service Planning Areas. These data were presented at 18 organizations throughout LAC and tailored to the organization’s catchment area and additional indictors of interest. Post-presentation discussions included data limitations and potential reasons for the results. Suggestions for future work included more granular data, expanded data sources (mortality rates and health service provider shortages), comparing age-adjusted rates, and stratifying geographic hospitalization rates by race/ethnicity. These health profiles generated important questions as to why there is high burden and poor management of disease and disability in some areas and how to develop interventions to address these issues at the local level.

## P21 Multiplying resources: impact and return on investment of 10 years of community-engaged research (CEnR) and partnership seed grants

### Melvin Thompson^1^, Rebecca Johnson^2^, Ivonne Kang^3^, Maryann Mason^2^, Gina Curry^2^

#### ^1^Endeleo Institute, Chicago, IL, USA; ^2^Northwestern University, Evanston, IL, USA; ^3^Apostolic Faith Church, Chicago, IL, USA

##### **Correspondence:** Melvin Thompson

Since its establishment in 2008, the Alliance for Research in Chicagoland Communities (ARCC) in the Northwestern University Clinical and Translational Sciences Institute (NUCATS) has spearheaded a seed grant program supporting collaborative research partnerships between Chicago communities and Northwestern. Over 10 rounds, the program has awarded a total of 51 grants, totaling more than $771K, that have led to over $10 million in external grants and 23 journal articles.

Grantees receive funds to build new partnerships or pilot research activities. A core goal is to build capacity and skills of communities and research teams to meaningfully engage at all stages of research. Collaborative data collection and analysis also facilitate competitive applications for external funding and continued partnership.

ARCC evaluates applicant experience and annually tracks outcomes. Data is collected from grantees, applicants, and reviewers, as well as university leadership and community engagement program staff. This evaluation has contributed to program evolution and improvement over the program’s successive rounds. This approach has also allowed ARCC to review institutional funding along with local health priority areas. For example, the most recent round encouraged applications focus on identified priorities of the Chicago Department of Public Health’s strategic plan and the Northwestern Memorial Hospital Community Health Needs Assessment.

Value-added features include a sustainable learning community for grantees, as well as increased understanding and appreciation of community-engaged research approaches in the Medical School at Northwestern and among community stakeholders. Awardees will describe program process and structure and grantee outcomes and impact from their unique perspectives.

## P22 Developing an institute-wide community advisory board at the University of Michigan Institute for Clinical and Health Research

### Karen Calhoun^1,2^, Zachary Rowe^3^, Dianne Carr^4^, Maria Thomas^5^, Ledon Charo^6^, Sarah Bailey^7^, Lisa Rentschler^5^, Kevin Weatherwax^8^, Tricia Piechowski^8^, Meghan Spiroff^8^, Adam Paberz^8^, Jorge Delva^8^, Tariq Madiha^9^

#### ^1^City Connect Detroit, Detroit, MI, USA; ^2^Michigan Institute for Clinical & Health Research, Ann Arbor, MI, USA; ^3^Friends of Parkside, Detroit, MI, USA; ^4^Ann Arbor YMCA, Ann Arbor, MI, USA; ^5^University of Michigan Hospitals and Health Center, Ann Arbor, MI, USA; ^6^IDE Buenos Vecinos, Ann Arbor, MI, USA; ^7^Community Member, Ann Arbor, MI, USA; ^8^Michigan Institute for Clinical & Health Research, Ann Arbor, MI, USA; ^9^Arab Community Center for Economic and Social Access, Dearborn, MI, USA

##### **Correspondence:** Karen Calhoun

The National Center for Advancing Translational Science (NCATS) requires hubs funded by the Clinical and Translational Science Awards (CTSA) to engage community partners in leadership, integration, and all phases of translational research. NCATS defines “community” broadly to include industry, patients, caregivers, and other stakeholders such as community-based organizations and community-based clinicians. In the past, the Michigan Institute for Clinical and Health Research (MICHR), the University of Michigan’s CTSA hub, engaged community partners through a Community Engagement (CE) Coordinating Council which provided guidance to the CE Program, not the full institute. This practice is common among CTSAs. A 2015 survey administered to CTSA CE programs reported the majority of CE programs have CABs. NCATS mandates that CTSAs utilize External Advisory Boards (EAB); however, EABs typically have minimal community partner representation. In 2014, a group of national community engagement experts recommended strengthening community partner leadership and inclusiveness across the institute. In response to this recommendation, a design team, comprised of diverse community stakeholders was created to recruit a CAB and draft its charter. To our knowledge, very few CTSAs include CABs to guide entire hubs. MICHR’s CAB will assist its leadership on matters such as program development, resource allocation, and policy to guide institutional priority setting. This study explored the process of developing the design team and the CAB and metrics used to evaluate both. We have developed CAB plans for ensuring communities are consulted for their research priorities and are respected, valued, and rewarded for their expertise.

## P23 Creating strong partnerships in the African-American community through honest and interactive educational events to SUPPORT and EMPOWER

### Audrey Farrow, Deborah Burcombe, Garrett Davis, Timothy Hughes, Laura Baker

#### Wake Forest School of Medicine, Winston-Salem, NC, USA

##### **Correspondence:** Audrey Farrow

Representation of older African Americans (AAs) in research on prevention and treatment of Alzheimer's disease (AD) is dismal, with estimates near 5% in NIH-funded AD trials. Traditional academic recruitment approaches often involve print advertising and impersonal messaging. This approach cannot address historic events that have damaged trust and perceptions of medical research. The science of community engagement in underserved communities emphasizes the importance of strong community partnerships that evolve from genuine, enduring, and mutually beneficial face-to-face interactions. We have developed a targeted Lunch-n-Learn three-part series for faith-based groups with primarily AA membership. The series includes 1) interactive discussions about normal and pathological cognitive aging, 2) the elephant in the room regarding past harmful medical research practices, changes in these practices, and the importance of research to ensure applicability to all, and 3) the impact of caregiving on the caregiver and his/her community. Since April 2016, 389 older adults have attended one of our 17 Lunch-n-Learn programs conducted in collaboration with eight large area churches. Of these, 102 AA adults have inquired about research opportunities (26%). This evolving partnership has also led to other collaborations, including the city-wide coalition of ministers, the Urban League, and the Black Repertory Theatre for which we provided audience Talk Backs following each performance of a nationally acclaimed play about AD. Successful community engagement requires acknowledgement of past atrocities and the development of a mutually beneficial relationship that provides support and fosters empowerment. Recruitment approaches that fail to address these needs are at high risk of failure.

## P24 Validated scale to measure the person-centeredness of research products

### Mckenzie Houston^1^, Ken Wallston^2^, Victoria Villalta-Gil^1^, Alan Richmond^3^, Yolanda Vaughn^4^, Sarah Stallings^1^, Consuelo H. Wilkins^1^

#### ^1^Meharry-Vanderbilt Alliance, Nashville, TN, USA; ^2^Vanderbilt University School of Nursing, Nashville, TN, USA; ^3^Community Campus Partnerships for Health, Nashville, TN, USA; ^4^Neighborhood Resource Center, Nashville, TN, USA

##### **Correspondence:** Consuelo H. Wilkins

**Background** Using data from community engagement studios and translational studios that collected project-specific input across a broad range of research areas, we developed a quantitative instrument to measure the patient-centeredness of research products.

**Materials and methods** A multi-step approach to scale development and validity included 1) content and item generation, 2) evaluation of item candidates, 3) testing of initial scale, 4) scale revision, and 5) testing of revised scale. Both community/patient stakeholders and researchers served as reviewers. Sixty research abstracts (RA) (30 PCORI RA and 30 ACTS RA) were rated with the first scale version (11 items, 4-point Likert scale). Feedback was also collected. The second version (seven items, 5-point Likert scale) was developed and tested using 40 RA (20 PCORI/20 ACTS). Factor analysis and Cronbach’s alpha were computed to determine internal consistency and reliability.

**Results** The first version internal consistency was high (alpha=0.930). Items showed correlation coefficients ranging from r=0.60 to r=0.86 with the scale total score. All items were included in one single factor explaining 59% of the variance. The second version showed high internal consistency (alpha=0.957), and items were highly intercorrelated (from r=0.63 to r=0.90). All items were included in one single factor explaining 80% of the variance. Mean score for PCORI RA was 7.15 (±7.96) and for ACTS RA was (-2.08±9.50).

**Conclusion** The quantitative Person-Centeredness of Research scale can be used by others in the field to help standardize this work and evaluate the patient-(person)-centeredness of research products.

## P25 Application of social network analysis for evaluating and improving partnership sustainability of local and statewide community health coalitions in Indiana

### Jennifer Mansfield, Lindley McDavid, Donna Vandergraff, Dennis Savaiano

#### Purdue University, Indianapolis, IN, USA

##### **Correspondence:** Jennifer Mansfield

The Indiana Clinical and Translational Sciences Institute (CTSI) Community Health Partnership (CHeP) community engagement model seeks to improve Hoosier health through community-university partnerships. At the county level, community health coalitions (CHCs) are central to improving health promotion partnerships and activities. However, the gamut of frameworks for evaluating CHC effectiveness limits comparisons between CHCs across inputs (e.g., partnerships, resources, context), activities, and health outcomes. Furthermore, CHCs logic models often assume that inputs explicitly lead to intended health outcomes, though rarely operationalize or evaluate partnership sustainability, which is a central mechanism to CHCs success. The application of social network analysis (SNA) is one methodology that could serve as the basis for a standardized evaluation and enable cross-sectional and longitudinal comparisons across CHCs for assessing key indicators of partnership sustainability (e.g., CHCs network robustness). SNA aligns with Community-Based Participatory Research principles by supplying formative feedback that informs CHC leaders about partnership structure and function. This feedback serves as the basis for recommendations that improve individual and statewide network health, including an evaluation of partnership sustainability. Additionally, we are integrating a cross-validation of multiple effectiveness indicators with SNA outputs in order to inform best practices for future CHCs programming and evaluation. As part of the CTSI CHeP, there are opportunities for statewide collaborative evaluations among Purdue Extension's community wellness coordinators, the Nutrition Education Program, SNAP-Ed, and the Indiana Healthy Weight Initiative. This novel approach to developing a standardized model for CHCs and comprehensive evaluation has great potential for contributing to community-engaged efforts to improve national health.

## P26 Violence as a health disparity: adolescents’ perceptions of violence depicted through photovoice

### Megan Irby^1^, Lynn Rhoades^2^, Nathan Ross Freeman^2^, DeWanna Hamlin^1^, Phillip Summers^1^, Scott D. Rhodes^1^, Stephanie Daniel^1^

#### ^1^Wake Forest School of Medicine, Winston-Salem, NC, USA; ^2^Authoring Action, Winston-Salem, NC, USA

##### **Correspondence:** Stephanie Daniel

Violence is a public health issue disproportionately affecting adolescents, particularly adolescents of color from vulnerable communities. In response to the growing concern of violence as a significant health issue, the Program in Community Engagement (PCE) at Wake Forest School of Medicine (WFSM) partnered with Authoring Action (A2), a community-based youth empowerment program, to better understand youth perceptions of violence in the community. This study used photovoice, a methodology aligned with community-based participatory research, to give adolescents a platform to voice their beliefs about violence. In 12 weekly sessions designed to foster an atmosphere of mutual trust and sharing, 10 adolescents from A2 engaged in research as both participants and data collectors. Adolescents received training in basic research methodologies (e.g., human subject protections and qualitative methods) and documented representations of violence through digital photography followed by crafting narratives to correspond with their photos. Facilitated by A2 and PCE staff, these youth engaged in empowerment-based photo-discussions focused on causes and consequences of violence, adolescents’ experiences with violence, and strategies to address violence. Adolescents and staff conducted a thematic analysis of photographs and narratives from which 18 themes emerged. Three primary themes were 1) violence stems from oppression, 2) cultural influences violence, and 3) the effects of violence on emotional and behavioral well-being. Health disparity and resilience were also prominent themes. Adolescents participated in a community forum to present their work to community stakeholders (e.g., law enforcement, health providers, and school personnel) in order to foster additional community discussions to address violence in the community.

## P27 Development and short-term impact of community research training curricula

### Lexie Lipham^1^, Jennifer Cunningham- Erves^2^, Yvonne Joosten^1^, Patrick Luther^3^, Stephania Miller-Hughes^2^

#### ^1^Vanderbilt University Medical Center, Nashville, TN, USA; ^2^Meharry Medical College, Nashville, TN, USA; ^3^Nashville CARES, Nashville, TN, USA

##### **Correspondence:** Lexie Lipham

**Introduction** The Meharry-Vanderbilt Community-Engaged Research Core (CERC) developed and piloted research training curricula (RTC) for community members (CM)s and community-based organizations (CBO)s. This work highlights the community-engaged RTC development process and pilot training results.

**Materials and Methods** Community partners recommended the need for RTC and were actively engaged in development via work groups, surveys, and meetings. Learning objectives and outlines were drafted and reviewed in two community engagement studios by CBOs and CMs. The RTC was piloted June - September, 2016. For piloting, pre-post questionnaires compared confidence in learning objectives, short-term impact on attitudes, and overall effectiveness. Two focus groups were conducted post-training to gather additional feedback. Descriptive statistics were used to analyze questionnaire results. Four raters independently coded and came to consensus on major focus group themes.

**Results** Community partners provided input into training content, frequency, and logistics for eight training sessions: one joint session for CMs and CBOs and separate sessions for CMs (n=2) and CBOs (n=5). Nine CMs and eight CBOs completed the training. The majority reported increased confidence (69%) and positive impact (72%) for each session. Major focus group themes were increased research empowerment and knowledge, increased desire to play a more meaningful role in their research endeavors, use of training materials in their professional and personal lives, and not enough time.

**Conclusion** Generally, the RTC positively impacted trainees and led them to feel more confident and empowered in research conversations and activities. Participant input allowed CERC to improve and standardize the training.

## P28 Community Scholars-in-Residence Program for graduate students and postdoctoral fellows: hands-on community-engaged research

### Karen Glanz, Sarah Green, Jill McDonald, Alyssa Yackle

#### University of Pennsylvania, Philadelphia, PA, USA

##### **Correspondence:** Jill McDonald

The Community Engagement and Research Core of the University of Pennsylvania (UPenn) CTSA launched a new Community Scholars-in-Residence (CSIR) Program to train young scholars in the science of community-engaged research. This two-year CSIR program is co-sponsored by the UPenn Prevention Research Center’s Cancer Prevention and Control Research Network (CPCRN). The program is designed to give young researchers hands-on experience in community-engaged health research and train them on best practices in the field.

The CSIR program’s goals are to introduce researchers to the fundamentals of community-engaged research early in their careers, train them on best practices, and help them develop the skills and mindset needed to conduct a successful research project and maintain positive relationships with community research partners. The selected Scholars will learn how to co-develop their respective research projects (in the area of Cancer Prevention and Control) with their community partners by participating in formal training sessions throughout the project period, receiving guidance and feedback from faculty mentors, and committing several hours each week to their community organizations. This program also aims to benefit the community partners by dedicating researcher time to shared initiatives.

An earlier, faculty-focused version of the CSIR program was adapted to be geared towards graduate students and postdoctoral fellows. Our expectation is that by teaching researchers the skills for community-engaged research early in their careers, they will be poised to incorporate community-engaged research methods into their work throughout their careers.

## P29 Engaging Latina breast cancer survivors in research: building a social network research registry

### Alejandra Hurtado de Mendoza^1^, Adriana Serrano^1^, Kristi Graves^1^, Nicole Fernandez^1^, Qu Zhu^1^, Valeria Massarelli^1^, Paola Rodriguez de Liebana^1^, Aileen Fernandez^1^, Claudia Campos^2^, Florencia Gonzales^3^, Tesha Coleman^4^, Laura Logie^2^, Vanessa Sheppard^5^

#### ^1^Georgetown University, Washington, DC, USA; ^2^Nueva Vida, Alexandria, DC, USA; ^3^Howard University, Washington, DC, USA; ^4^Capital Breast Care Center, Washington, DC, USA; ^5^Virginia Commonwealth University, Richmond, VA, USA

##### **Correspondence:** Alejandra Hurtado de Mendoza

**Background** Latinos are underrepresented in research, and the aim of this study is to develop an innovative Social Network Research Registry to enhance research engagement of Latina breast cancer survivors.

**Materials and methods** We recruited 29 Latina breast cancer survivors from two community organizations (the seeds). Using cascade recruitment, we asked the seeds to identify other Latina breast cancer survivors. Using social network measures we captured the structural (e.g., size) and functional characteristics (e.g., social support) of the network of Latina survivors. We invited participants to be part of the Registry, to be contacted for future studies, and to specify the types of research and engagement.

**Results** We recruited 50 participants in four months (29 seeds, 21 through snowballing). All agreed to be part of the Registry. Participants listed a range of 0-11 Latina survivors (*Mdn*=3). The total network size is 107 and is formed by six components. We have identified four very highly connected survivors (hubs). The most available type of perceived support was companionship (*Mdn* =3) and provision of information about breast cancer (*Mdn* =3). All were interested in participating in surveys or interviews and most in behavioral interventions (95%), providing biological samples (88%), and drug trials (33%). Most were interested in being engaged in research as health promoters (81%) or members of the community advisory board (67%).

**Conclusion** Social network analysis can be useful for identifying isolated participants and members occupying key positions in the network who can be engaged to inform future research and disseminate health information.

## P30 A community-engaged approach to measuring trust in biomedical research

### Nicollette Davis^1^, Jacquelyn Favours^2^, Sarah Stallings^2^, Consuelo Wilkins^2^

#### ^1^Vanderbilt Institute for Clinical Translational Research, Nashville, TN; ^2^Meharry-Vanderbilt Alliance, Nashville, TN

##### **Correspondence:** Consuelo Wilkins

**Background** Lack of trust toward research is one of the most commonly cited barriers to study participation, especially among groups underrepresented in research [1-2]. Of 45 instruments for measuring trust, only two are related to biomedical research [3], and neither of the two includes the four trust areas most commonly identified by racial/ethnic minorities [4-6]. The objective of this study is to understand elements of trust in research among underrepresented groups that may not be reflected in existing trust scales.

**Materials and methods** We conducted a series of seven focus groups, taking a cross-cultural approach with three racial/ethnic groups. Participants were consented and completed a demographic survey and either the Hall or Mainous Trust Scale. Topics discussed included Research, Trust, Privacy/Confidentiality, and Research Participation. Transcripts of the focus groups were blindly reviewed, and excerpts were coded for themes by two independent coders.

**Results** Of the 58 participants, 80% were racial/ethnic minorities, 69% had no prior research participation, 39% had an education level of high school diploma or less, and 33% had an annual household income of less than $15,000. In preliminary thematic analysis, results show that trust in research varies based on the research institution’s history, time spent by the researcher on enrollment, completeness of study information given, and recruitment appeal source (i.e., friend, doctor, respected community figure, flyer, radio, etc.).

**Discussion** Perspectives on trust among racial/ethnic minorities and individuals with limited income differ from the majority population and may not be captured by existing trust measures. New or adapted measures of trust are needed to assess trust among these groups.

**References**

1. Scharff DP, Mathews KJ, Jackson P, Hoffsuemmer J, Martin E, Edwards D. More than Tuskegee: understanding mistrust about research participation. J Health Care Poor Underserved. 2010: 21:879-897.

2. George S, Duran N, Norris K. A systematic review of barriers and facilitators to minority research participation among African Americans, Latinos, Asian Americans, and Pacific Islanders. Am J Public Health. 2014: 104:e16-31.

3. Ozawa S, Sripad P. How do you measure trust in the health system? A systematic review of the literature. Soc Sci Med. 2013: 91:10-14.

4. Durant RW, Legedza AT, Marcantonio ER, Freeman MB, Landon BE. Different types of distrust in clinical research among whites and African Americans. JNMA 2011:103:123-30.

5. Armstrong K, McMurphy S, Dean LT, et al. Differences in the patterns of healthcare system distrust between blacks and whites. J Gen Int Med. 2008: 23:827-833.

6. Boulware LE, Cooper LA, Ratner LE, LaVeist TA, Powe NR. Race and trust in the health care system. Pub Health Rep 2003:118:358.

## P31 Creating a strategic alliance with diverse partners to address health disparities through innovative precision health research

### Jill Evans^1^, Rhonda McClinton-Brown^1^, Van Ta Park^2^, Ysabel Duron^3^, Jan Vasquez^4^, Owen Garrick^5^

#### ^1^Stanford University Center for Population Health Sciences, Stanford, CA, USA; ^2^University of California, San Francisco, San Francisco, CA, USA; ^3^Latinas Contra Cancer, San Jose, CA, USA; ^4^Timpany Center, Fruitdale, CA, USA; ^5^Bridge Clinical Works, San Francisco, CA, USA

##### **Correspondence:** Jill Evans

Precision health takes into account people’s individual differences in genes, environment, and lifestyle to address disease prevention and treatment. Accordingly, precision health offers a new paradigm for optimizing population health through genuine partnership with patients, providers, community organizations, and key stakeholders. In 2016, Stanford Precision Health for Ethnic and Racial Equity (SPHERE) was launched as one of the first national centers funded by the National Institute of Minority Health and Health Disparities to focus on using precision medicine tools to improve the health of underserved ethnic and racial groups. SPHERE’s transdisciplinary approach to precision health calls for innovative engagement models that can be adapted to the complexities of the research projects and the various stakeholder groups. Initiatives in the SPHERE Consortium and Implementation Cores inform effective approaches to engage key and underserved population groups to maximize the potential of precision health in reducing health disparities. The innovative approaches employed by SPHERE combine strategies from CBPR, patient-centered research, and Team Science to optimize engagement in the development, design, testing, and delivery of precision health approaches. This flexibility and agility in engagement aim to increase the ease and willingness of diverse stakeholders to remain highly engaged over the course of the 5-year project.

## P32 Assessing organizational capacity needs to promote partnership readiness for community-engaged research

### Hilary Broughton^1^, Mei-Hsi Chiang^2^, Sarah Bobmeyer^2^, Angela Brown^2^

#### ^1^Washington University School of Medicine, St. Louis, MO, USA; ^2^Washington University in St. Louis, St. Louis, MO, USA

##### **Correspondence:** Hilary Broughton

**Background** The objective of this study is to describe methods used to conduct an organizational capacity needs assessment with community-based organizations (CBOs) in the St. Louis region.

For community-engaged research to be equitable, effective, and sustainable, all partners must be ready for collaboration and shared leadership responsibilities. Thus, a core value of community-engaged research is the opportunity for partners to build capacity. In order to frame efforts and catalyze partnership readiness, we must better understand the current capacity and needs of CBOs.

**Materials and methods** Through a collaborative process, we 1) met with stakeholders to finalize the initiative purpose and approach, 2) developed an assessment tool and interview guide based on existing tools and stakeholder feedback, 3) conducted the assessment and interviews with a sample of CBOs, and 4) provided an aggregate report and individualized reports to participants.

**Results** Stakeholders identified six capacity areas as essential for partnership readiness. Ninety-four participants representing 49 organizations (83% response rate) completed the assessment, and 20 participants completed a qualitative interview. Priority training needs identified include 1) program evaluation, 2) data management, 3) leadership development, 4) communication strategy development, and 5) human resources management. The report frames the most effective ways to address capacity needs and development of capacity-building funding opportunities.

**Conclusion** Assessing capacity is critical in promoting balanced partnerships. CBOs identified needs in areas that university partners can support. Ultimately, this builds relationships, enhances research effectiveness, and supports sustainability of promising interventions.

## P33 PCORnet obesity observational study: Short- and long-term effects of antibiotics on childhood growth - gathering stakeholder feedback to improve engagement process

### Andrea S. Goodman^1^, Anthony Solomonides^2^, Ivette C. Torres^3^, Jordan Capizola^4^, Kathleen Murphy Marks^5^, Juliane S. Reynolds^6^, Jason P. Block^7^, Sharon F. Terry^8^, Doug Lunsford

#### ^1^Genetic Alliance, Washington, DC, USA; ^2^NorthShore University HealthSystem Research Institute, Evanston, IL, USA; ^3^Rio Grande Valley Breastfeeding Coalition, Harlingen, TX, USA; ^4^Genetic Alliance, Washington, DC, USA; ^5^Genetic Alliance, Washington, DC, USA; ^6^Therapeutics Research and Infectious Disease Epidemiology Group, Department of Population Medicine, Harvard Pilgrim Health Care Institute, Harvard Medical School, Harvard University, Boston, MA, USA; ^7^Division of Chronic Disease, Research Across the Lifecourse, Department of Population Medicine, Harvard Pilgrim Health Care Institute, Harvard Medical School, Harvard University, Boston, MA, USA; ^8^Genetic Alliance, Washington, DC, USA; ^9^North Fork School District, Utica, OH, USA

##### **Correspondence:** Kathleen E. Murphy

**Background** Using an innovative, multi-site network called PCORnet, the PCORnet Antibiotics Study utilizes electronic health record data from 37 healthcare institutions across the nation. The study explores the effects of antibiotic use during the first two years of life on BMI at ages 5 and 10.

An Executive Antibiotics Stakeholder Advisory Group (EASAG) was formed to work alongside the scientific team to carry out the study’s objectives. Members include parents, caregivers, pediatricians, pharmacists, investigators, and advocacy/health systems leaders.

**Materials and Methods** In March 2017, EASAG facilitators surveyed stakeholders to evaluate their involvement and engagement. Goals included 1) determining stakeholder satisfaction, 2) informing the study’s engagement process, and 3) documenting lessons learned to benefit future studies.

EASAG members reviewed the draft survey and provided feedback prior to finalization.

**Results** The survey garnered a 100% participation rate. Respondents reported feeling satisfied with their engagement. More than half reported having some decision-making authority and ability to contribute meaningfully to study objectives. All respondents agreed stakeholders engaged in open, respectful communication. Some stakeholders indicated their expertise could be better utilized, while some were unsure how to best lend their skills to support the study.

**Conclusion** The high response rate may have been due to the participatory process of developing the survey. Findings will be shared broadly to inform stakeholder inclusion in scientific studies, sustain engagement, and enhance future PCORnet stakeholder engagement. Special consideration and effort will be devoted to better integrating stakeholders’ technical skills and ensuring all stakeholders feel they have meaningfully contributed.

## P34 Are social determinants of trust determined by our choice of trust measurements?

### Jennifer Cunningham- Erves^1^, Victoria Villalta-Gil^2^, Alecia Fair^2^, Jacquelyn Favours^2^, Rowena Dolor^3^, Duane Smoot^1^, Consuelo H. Wilkins^2^

#### ^1^Meharry Medical College, Nashville, TN, USA; ^2^Meharry-Vanderbilt Alliance, Nashville, TN, USA; ^3^Duke Clinical Research Institute, Raleigh, NC, USA

##### **Correspondence:** Jennifer Cunningham- Erves

**Background** Public participation in research, especially among underrepresented populations, is commonly impeded by lack of trust towards research. Assessing trust towards research is important, and few validated measures exist. It is unknown if different variables will predict the scores in different validated trust scales. We aimed to determine if choice of trust scale will shape different sociodemographic determinants of trust.

**Materials and methods** This cross-sectional study had a total of 3,753 adults (565 African-American (AAP)/3,188 Caucasian (CP) adults) randomly assigned to complete one of two surveys, which were identical other than including one of two scales assessing trust in medical research (1,2). The surveys also assessed willingness to participate in research and barriers to participation. Linear regression was used to determine predictors of trust in both trust measures.

**Results** 1,906 participants completed the HTS, and 1,847 completed the MTS. Including race as a covariate changed the model accuracy from 10.1% to 11.2% for the HTS and from 14.8% to 21.3% for the MTS. Scores of the HTS were predicted (R=0.294, F=28.75***) by barriers to research participation (β=-0.067), race (β=0.38), education (β=-0.063), health literacy (HL) (β=0.028), and health numeracy (HN) (β=0.010). Scores of the MTS were predicted (R=0.445, F=73.53***) by race (β=0.110), HL (β=0.51), TB (β=-0.061), HN (β=0.020) and age (β=-0.002).

**Conclusion** Despite differences in the relative importance of trust determinants, there was overlap between the scales: race, health literacy, health numeracy, and barriers to research participation were relevant predictors of trust measures. When designing interventions to improve trust, we should consider these sociodemographic factors.

**References**

1. Hall MA, Camacho F, Lawlor JS, DePuy V, Sugarman J, Weinfurt K. Measuring Trust in Medical Researchers. Med Care. 2006;44(11):1048–1053.

2. Mainous AG, Smith DW, Geesey ME, Tilley BC. Development of a Measure to Assess Patient Trust in Medical Researchers. Ann Fam Med. 2006 May 1;4(3):247–252.

## P35 A tailored educational program to improve cancer clinical trial participation among African Americans and Latinos

### Jennifer Cunningham-Erves^1^, Claudia Barajas^2^, Tilicia Mayo-Gamble^3^, Caree R. McAfee^4^, Pamela Hull^4^, Maureen Sanderson^1^, Juan Canedo^1^, Katina Beard^5^, Consuelo H. Wilkins^6^

#### ^1^Meharry Medical College, Nashville, Tennessee, USA; ^2^Vanderbilt-Ingram Cancer Center, Nashville, TN USA; ^3^Georgia Southern University, Statesboro, GA, USA; ^4^Vanderbilt University Medical Center, Nashville, TN, USA; ^5^Matthew Walker Comprehensive Health Center, Nashville, TN, USA; ^6^Meharry Vanderbilt Alliance, Nashville, TN, USA

##### **Correspondence:** Jennifer Cunningham-Erves

**Background** Participation in cancer clinical trials is low in minorities, particularly among African Americans and Latinos. Lack of awareness and knowledge regarding clinical trials are major barriers to participation. The objective of this study is to develop a tailored educational program to increase awareness and understanding of cancer clinical trials.

**Materials and methods** We used an iterative, community-engaged adaptation process to create culturally and linguistically appropriate content for African Americans and Latinos. We first identified an existing clinical trials education program and held a focus group with peer educators who previously delivered the program. Next we conducted nine focus groups (four with African Americans, five with Latino) (N=85) to obtain input on ways to improve the educational program. We then revised the program based on feedback. The community review board and the researcher reviewed the data summary and finalized the educational program.

**Results** The key focus group findings were used to identify and integrate content related to cancer (e.g., definition, risk factors, statistics such as top cancer deaths by race and gender) and clinical trials (e.g., definition, process, participation costs, and clinical trial resources). Minor changes were also made in content, length (i.e., shorter), color scheme (i.e., lighter tones), and visual aids. We also developed testimonials from the African-American and Latino researcher and community member perspectives on cancer clinical trial participation.

**Conclusion** The tailored clinical trial educational program includes content and format deemed more appropriate and relevant to African Americans and Latinos. The new program will be delivered in community settings and compared to the untailored version to determine impact on knowledge, awareness, and willingness to participate in clinical trials.

## P36 A community organization assessment for identifying barriers to data dissemination for community-based participatory research (CBPR) findings in a faith-based community: the Washington, DC, cardiovascular health and needs assessment

### JaWanna L. Henry^1^, Dana M. Sampson^2^, Tiffany Powell-Wiley^3^

#### ^1^Office of the National Coordinator for Health Information Technology, Washington, DC, USA; ^2^Office of Minority Health for the U.S. Department of Health and Human Services, Rockville, MD, USA; ^3^Cardiovascular and Pulmonary Branch of the Division of Intramural Research, National Heart, Lung and Blood Institute, National Institutes of Health, Bethesda, MD, USA

**Background** Early data dissemination correlates to sustained health improvements in Community-Based Participatory Research (CBPR). However, little is known about the utility of assessing barriers to data dissemination in CBPR study implementation. This project assesses community organization (CO) for planning data dissemination within at-risk neighborhoods for cardiovascular health advancement.

We assessed CO constructs (empowerment, community capacity, social capital) as communication barriers for data dissemination from a cardiovascular health and needs assessment among faith-based organizations in at-risk Washington, DC, neighborhoods (NCT 01927783). This CO evaluation was a first step to develop iterative CBPR project data dissemination plans.

**Materials and methods** Community members from partnering Washington, DC, churches (n=24) attended one of three meetings to 1) receive dissemination of early findings from the health and needs assessment, 2) complete a survey measuring CO constructs using validated scales (higher score=greater CO), and 3) provide focus group data on CO topics and preferred data dissemination methods. Mean scores were determined for each CO scale. Qualitative data were analyzed for proposed dissemination barriers and solutions.

**Results** Participants were 96% African-American and 79% women (ages=39-79 years). Scores for empowerment, community capacity, and social capital were mean [standard deviation (SD)] 34.5(3.5)(max=48), 20(3.4)(max=30) and 7(1.9)(max=10), respectively. During the group-based discussion, participants described difficult resource mobilization as a data dissemination barrier.

**Conclusion** Qualitative and quantitative findings suggest community capacity as a barrier to CBPR data dissemination in a predominantly African-American, faith-based Washington, DC, community. Data dissemination efforts using methods proposed by community members, including written materials, web-based platforms, and in-person forums, may enhance community capacity for future CBPR success.

This conference series used novel approaches to fulfill its objectives of convening a diverse group of participants, meaningfully engaging stakeholders, and eliciting diverse perspectives to advance the science of community engaged research. Innovative strategies for the conference included 11 Learning Labs that offered participants unique opportunities to gain practical knowledge regarding innovative methods in community engaged research. Learning Labs allowed new approaches to be rapidly disseminated with the goal of speeding implementation of community engaged methods.

## LL1 Implementing a community-patient scientist academy to engage underrepresented populations in research

### Kate Stewart, Anna Huff Davis

#### Translational Research Institute University of Arkansas for Medical Sciences, Little Rock, AR, USA

##### **Correspondence:** Kate Stewart

**Learning Objectives:** By the end of the lab, participants will be able to 1) List the two main objectives of the community / patient scientist academy, 2) Articulate at least three key concepts covered in the academy, 3) Describe at least two interactive exercises used to engage participants in the academy

Community members and patients often feel intimidated by research and may have difficulty seeing the importance of their role as research participants and/or advisors and partners in the research process. When community engagement (CE) leaders of the Translational Research Institute (TRI) at the University of Arkansas for Medical Sciences asked their Community Advisory Board (CAB) how to address this challenge, they recommended we implement a community-patient scientist academy (CPSA). Through this learning lab, members of our CE academic-community team sought to show participants how to implement a CPSA based on the model they developed with TRI CAB and CE staff members.

The CPSA is designed to engage community members and patients who are underrepresented in research, have no research background, and who may lack trust and interest in participating in research. Objectives of this 10-hour, 5-week introductory course about research are to 1) increase community members’ and patients’ understanding about the research process and 2) increase access to opportunities for them to influence and participate in research. Content of sessions includes basic information about 1) research definitions, the research process, different types of research, research partnerships, how research questions are formed, 2) study design, 3) the grant review process, 4) study implementation and dissemination, and 5) ways to be involved in the research process and in the TRI. A sixth session is held for the graduation ceremony. In session five, participants are given the opportunity to indicate their interest in being involved in TRI or other research activities (e.g., community review boards, patient and family advisory councils, reviewing grants, etc.). Sessions include a brief presentation of key concepts followed by one or two guest researchers invited to discuss their research, focusing on the key concept for the session (e.g., study design, implementation, etc.). Researchers share informally in small groups of participants, explaining their research, and answering questions. Key concepts from the previous week are reviewed each week using interactive exercises. A pre- and post-knowledge and feedback survey is administered each week to evaluate learning and to identify ways to improve the course. CPSA graduates are now participating in TRI’s research-related activities.

Learning lab presenters give a mini-session demonstration and engage participants through illustrative interactive exercises. Session leaders will walk through the CPSA toolkit and guide participants in a problem solving exercise addressing barriers and challenges they might face in implementing the CPSA.

## LL2 Best-practice strategies for engaging community stakeholders and patients as partners in research

### Tilicia L. Mayo-Gamble^1^, Velma McBride Murry^2^

#### ^1^Georgia Southern University, Statesboro, GA, USA; ^2^Vanderbilt University, Nashville, TN, USA

##### **Correspondence:** Tilicia L. Mayo-Gamble

**Learning Objective:** Participants will be able to identify effective strategies for engaging community stakeholders and patients as partners in research with an emphasis on expectations for challenges and strengths.

Community engagement and community-engaged research are viewed as the cornerstones of improving health and reducing health disparities in underserved and underrepresented communities. While the importance of community-engaged research is increasingly recognized as a critical component to improving patient-care and reducing health disparities, for researchers new to this approach for conducting research, deciding how to establish community partnerships can be a challenge. This learning lab provides tools to facilitate implementation of best practice strategies for engaging community stakeholders and patients as partners in research. A learning lab was held to teach participants how to implement a basic, introductory research academy for community members and patients. Learning how to use this resource is highly relevant to community-engaged research due to its potential for bridging the gap between researchers and those they seek to engage. Through personal contact with researchers and by creating a safe space for exposure to concepts presented in lay language using interactive exercises, this tool can demystify a process that is often highly intimidating to those not involved in research. In addition to increasing the likelihood for future engagement as participants in research, the academy can create a pool of community members and patients who can be called on to be involved in research in deeper ways (e.g., through community advisory and/or review boards, as community grant reviewers, in recruiting others to research, or as research partners or co-investigators).

## LL3 Sharing research results with those who need them: engaging with community partners to plan effective disseminations

### Rachel Hemphill^1^, Lisa Stewart^1^, Vanessa Ramirez-Zohfeld^2^

#### ^1^Patient-Centered Outcomes Research Institute, Washington, DC, USA; ^2^Northwestern University, Evanston, IL, USA

##### **Correspondence:** Rachel Hemphill

**Learning objectives**: 1) to learn about methods for working with community partners to plan and prepare for effective dissemination of study results to end-users, 2) to generate ideas for a dissemination plan for a research case study, and 3) to identify challenges for getting study results to end-users and share potential solutions and lessons learned

Getting results from clinical research into the hands of patients and other end-users (e.g., caregivers, clinicians, policymakers, insurers) is crucial for improving the healthcare system and people’s ability to make informed decisions about their health. This requires more innovative methods of community engagement. Traditional methods of disseminating research results, such as publications in academic journals, are typically not accessible or tailored to many of the audiences who could benefit from the information. This learning lab will focus on ways to partner with patients and other communities throughout the research process to plan and prepare for effective dissemination of results to diverse audiences. Speakers presented key elements from PCORI’s Dissemination and Implementation Framework and shared examples from PCORI-funded studies of partner involvement in dissemination activities. Through interactive activities, participants considered key questions to inform an effective, multifaceted dissemination plan and problem-solve about challenges that may arise as study teams plan disseminations and share results with end-users. This learning lab is relevant for researchers who are currently conducting or planning a community-engaged research study, as well as patients and other community members engaged as research partners.

## LL4 Strategies for engaging the community in creating patient-centered research questions

### Shivonne Laird, Courtney Clyatt

#### Patient-Centered Outcomes Research Institute, Washington, DC, USA

##### **Correspondence:** Shivonne Laird

**Learning objectives** :1) understanding what makes a good patient-centered research question, 2) learning what type of information can be used to inform a patient-centered research question and how anyone (including patient and community groups) can collect this information, 3) learning how researchers can use data to make their research question patient-centered or community-relevant, and 4) discussing ways patients and community members can engage with researchers, and vice versa, to ensure research questions are relevant.

To make healthcare research more relevant to patients/caregivers and other stakeholders, especially in marginalized and under-resourced communities, it is important that studies are designed to produce results that may be useful to them. This learning lab was conducted to share strategies for creating or helping to create a research question that is patient-centered. With strategies drawn from the Patient-Centered Outcomes Research Institute (PCORI) Eugene Washington PCORI Engagement Awards program, as well as the Pipeline to Proposal program and research portfolio, this session shared practical steps for creating a patient-centered research question, with tips for researchers, patients, and other stakeholders in healthcare.

## LL5 Mile-high community engagement: developing a training pipeline for community-based participatory researchers in Colorado

### Victoria Francies, Mary Fisher, Montelle Tamez

#### Colorado Clinical and Translational Sciences Institute, University of Colorado, Denver, Denver, CO, USA

##### **Correspondence:** Mary Fisher

**Learning Objectives:** 1) describe how the pipeline within community engagement for researchers and community members can enhance CBPR research practice and increase community participation and capacity and 2) learn how to incorporate the roles of Community Research Liaisons and create Immersion Programs for community engagement.

The Colorado Clinical Translational Sciences Institute (CCTSI) Community Engagement Core at the University of Colorado, has developed a pipeline model for researchers and community to build capacity for community-based participatory research faculties. The pipeline includes Community Research Liaisons, the Colorado Immersion Training, Partnership Development and Joint Pilot Grants, and the Community Consults and Ethics Committee. All activities within the CCTSI Community Engagement are under the oversight and direction of the Partnership of Academicians and Communities for Translation (PACT) Council. The PACT Council is comprised of seven academic and seven community partners.

Researchers can apply to receive training and support in CBPR and community-engaged research from the CCTSI. This support comes in the form of the Colorado Immersion Training, which introduces academic researchers to communities within specific geographic and demographic groups in Colorado. Once Colorado Immersion Training is completed, researchers are supported and encouraged to apply for Partnership Development Grants through CCTSI Pilot Grants. These grants fund time dedicated to building relationships between research and community. Grantees will receive hands-on coaching and mentorship with community research liaisons and are encouraged to continue CBPR practices in their research. After completing their Partnership Development grant, they can apply for another 12 months of funding to begin research through a joint pilot grant. Both grant mechanisms require at least 50% of funds to go to the community. The next portion of the pipeline includes direct consultation with a community-based Consults and Ethics Committee, providing direction and processes to ethically include community members in research. Research participants in the pipeline are encouraged to stay connected and can even consider applying to become a part of the PACT Council, fully completing the pipeline.

This session highlighted the successes of the CCTSI Community Engagement Pipeline and encouraged discussion and peer support around community-engaged activities. CCTSI Staff shared best practices and results from current programs and grant activities, allowing participants to gain valuable insight into successful community engagement activities in an established academic setting.

## LL6 Engaging diverse communities to understand how precision health research can address health disparities

### Lisa Goldman Rosas, Rhonda McClinton-Brown, Jill Evans

#### Stanford University, Stanford, CA, USA

##### **Correspondence:** Lisa Goldman Rosas

**Learning objectives:** 1) discuss the barriers and facilitators of developing and implementing precision health research in diverse racial/ethnic communities, 2) identify best practices for developing community-university partnerships for precision health research, 3) understand how to develop and implement research to engage diverse communities in precision health research, 4) identify best practices for working with researchers from diverse disciplines to incorporate community engagement in their research, 5) become familiar with existing resources for increasing communities’ capacity for engaging in precision health research, and 6) discuss diverse community’s understanding and perception of precision health research and related best practices for implementation of precision health research.

The goal of this learning lab was to provide participants with skills, resources, and best practices for engaging diverse community groups in precision health research aimed at addressing health disparities. The learning lab was based on our experience as part of the Stanford Precision Health for Ethnic and Racial Equity (SPHERE) project. The goal of SPHERE is to promote effective dissemination and adoption of precision health approaches to ameliorate health disparities. Precision health includes disease prevention and treatment for maintenance of health and wellness across the life course that is proactive, predictive, effective, efficient, and equitable. Precision health holds great potential for revolutionizing health and healthcare through a better understanding of the complex interplay between biological, behavioral, environmental, and social factors that contribute to health inequalities and influence population health. However, the precision health movement may widen disparities if barriers (e.g., socioeconomic, language, racial/ethnic) prevent health disparities populations from being included in research and gaining access to precision health approaches that develop from this research.

Engagement of racial/ethnic minority communities is critical for informing and testing relevant and effective precision health strategies that will address health disparities. However, barriers to engaging racial/ethnic minority communities in precision health research include researchers’ lack of experience engaging the community in this topic, low awareness/knowledge of precision health among community members, conflicting priorities in low resource settings, and ethical and trust concerns. As a first step in addressing these barriers, we developed a community-university partnership with five organizations to engage diverse racial/ethnic communities and the providers that serve them. To develop the partnership, we collaboratively designed a study to gain a deeper understanding of perceptions of precision health, potential for precision health research to address health disparities, willingness to participate in precision health research, and ideas for increasing awareness about precision health research in racial/ethnic minority communities. We conducted 12 focus groups with five racial/ethnic minority communities (Vietnamese, Chinese, American Indian, Latino, African-American) and providers in English and, if relevant, in the native language. During the learning lab, we discussed 1) best practices in developing partnerships for precision health research, 2) skills for designing a qualitative study that is able to obtain input from six diverse communities, 3) resources for increasing awareness about precision health research in diverse communities, 4) methodology for analyzing data from diverse community groups, and 5) similarities and differences in results across the six groups.

## LL7 Maximizing value of stakeholder engagement: tips and tools from stakeholder engagement consulting on nine PCORI-funded studies

### Gay R. Thomas, Betty L Kaiser

#### Wisconsin Network for Research Support, University of Wisconsin-Madison School of Nursing, Madison, WI, USA

##### **Correspondence:** Gay R. Thomas

**Learning objectives:** 1) identify orientation activities that prepare stakeholders to effectively participate in the project, 2) recognize elements of a stakeholder meeting agenda that can yield constructive feedback for the research team, and 3) describe key strategies to sustain stakeholder engagement across the project lifespan.

Many researchers who want to involve stakeholders in community-engaged research (CeNR) can readily identify those they want as stakeholders on their projects. Identifying key stakeholders is a necessary step, but it is not sufficient to ensure effective, sustainable stakeholder engagement that produces valuable outcomes for the research team. In our four years of experience as stakeholder engagement consultants on nine PCORI-funded projects and an equal number of other patient-centered research studies, we have seen that researchers often begin community-engaged research with great enthusiasm but without a clear conception of the detailed steps involved in successful stakeholder engagement. In the absence of a comprehensive plan, stakeholder engagement can falter, and the outcomes of engagement can be disappointing both for researchers and stakeholders. In our role as consultants, we have observed several problems related to incomplete planning:

- Roles for stakeholders are not clearly defined.
- Stakeholders are not appropriately prepared for their work.
- In projects with multiple stakeholder groups, the research team does not have a transparent and consistent process for synthesizing feedback from all groups.
- Stakeholder meeting agendas are not intentionally constructed to support meaningful stakeholder participation and instead promote passive listening to reports from the research team.

We have developed a specific process and templates to help researchers anticipate and address these issues. This learning lab provided participants with practical tips and tools to maximize the value of work with research project stakeholders. The content is most appropriate to researchers who want to work with patient and community stakeholders, including those from under-represented populations.

## LL8 The forgotten stakeholder: partnering with university administrators to create compensation and recognition mechanisms that support efficiency, fairness, and sustainability in community engagement

### Lori Carter-Edwards, Ginny Lewis

#### NC Translational and Clinical Sciences Institute, University of North Carolina at Chapel Hill, Chapel Hill, NC, USA

##### **Correspondence:** Lori Carter-Edwards

**Learning Objectives:** To enable participants to 1) define efficiency, fairness, and sustainability in CEnR stakeholder engagement from the perspectives of a) health providing/seeking communities, b) academic researchers, c) research grant administrators, and c) university administrators; 2) discuss categories of university mechanisms for recognition and compensation of non-employee stakeholders in health research, as well as other non-financial compensation benefits for stakeholders; and

3) develop strategic plans to build and strengthen efficiency, fairness, and sustainability in stakeholder engagement initiatives that utilize one or more of the university mechanisms for recognition and compensation of non-employee stakeholders in health research.

Creating community-academic research partnerships for community-engaged research (CEnR) initiatives among stakeholders from underrepresented and underserved populations should involve equitable collaborations at all stages, including modes for compensation and recognition. However, university compensation of community partners is often nebulous and unstructured with no clear mechanism for preparing them as independent contractors for CEnR and appropriately recognizing them for their time and expertise. The North Carolina Translational and Clinical Sciences Institute (NC TraCS) at the University of North Carolina at Chapel Hill (UNC-CH) launched its Community-Academic Grants Administration Translation (CAGAT) training initiative in 2016 to adapt academic institutional protocols and procedures to facilitate CEnR for community partners and researchers. In 2017, as a follow-up on this initiative, CEnR faculty and staff at NC TraCS adapted the UNC-CH’s bidding process for exterior contractors to create an ongoing cycle of community stakeholder recruitment, capacity building, and compensation. Essential to this translation of institutional business platforms for CEnR was the engagement of UNC-CH purchasing and compliance officials who led CEnR faculty and staff through a rapid acculturation to the mores and standards of university fiduciary administration. These officials (often the forgotten stakeholders who should be recognized as part of our stakeholder community in building CEnR protocols) contributed their expertise in managing a compensation infrastructure for non-traditional university business partners.

Questions of efficiency, fairness, and sustainability in the compensation of independent contractors drove the development of a new NC TraCS community stakeholder engagement compensation structure. Given the amount of time-intensive relationship-building required of investigators, as well as of community partners, to create CEnR partnerships, working with experienced stakeholders on multiple projects can result in greater ease of engagement that can be interpreted as efficient by investigators and research professionals with limited time. While experienced or professional stakeholders develop a deeper level of understanding of the research process than most community partners with whom the university has not engaged, there were questions of fairness to community members who did not have entry to the initial recruitment of stakeholders for research consultations. One key issue involved balancing the efficiency of repeating stakeholder partnerships on multiple research projects with fairness and long-term sustainability for community stakeholders. Purchasing and compliance officials at UNC-CH shared their expertise in balancing these questions within university business practices and, in turn, engaged in the development of CEnR guidelines and practices that can serve other academic institutions.

## LL9 Development, implementation and evaluation of a community-engaged advisory board: best practices for strategies for maximizing success

### Alicia Matthews, Amaparo Castillo, Emily Anderson

#### University of Illinois at Chicago, Chicago, IL, USA

##### **Correspondence:** Alicia Matthews

Since 2006, the National Institutes of Health have supported the Centers for Translational Sciences Awards (CTSAs). These programs are designed to improve the health of individuals and the public by developing innovative approaches to translating basic science findings in the laboratory to clinical and community interventions. Community engagement is essential to the successful translation of interventions and other healthcare advances implemented in community settings. Researchers who work with community-based providers and special populations are aware of the need to involve them throughout intervention development and testing. However, less attention has been devoted to the inclusion of communities in the process of setting research priorities and engaging communities to advise researchers at all stages of the translational continuum.

In this learning lab, our aim was to highlight best practices in the development, implementation, maintenance, and evaluation of a long-standing Community Engagement Advisory Board (CEAB) at the University of Illinois at Chicago (UIC). The UIC CEAB is a resource of the Recruitment, Retention, and Community Engagement consultation service of the university’s CTSA investigators and community representatives. The chief purpose of the CEAB is to provide support and advisement for researchers at UIC. The CEAB gives advice and input on promoting a community-responsive research agenda reflecting a broad definition of health that acknowledges the interrelationships among individual, social, environmental, political, and economic determinants of health. The role of the CEAB members is to provide feedback to consultation recipients on any number of research issues in which the consultation recipient seeks input from the board members. This model can be used to inform the development of future CEAB boards.

## LL10 Helping community members claim their power: building capacity to partner with research institutions

### Tiffany Israel, Alexis Gorden, Yvonne Joosten

#### Vanderbilt University Medical Center, Nashville, TN, USA

##### **Correspondence:** Yvonne Joosten

**Learning Objectives:** 1) identify potential roles for patients and other community stakeholders as active partners with research institutions, 2) develop knowledge of essential elements for increasing community capacity to take on meaningful roles with research institutions, and 3) identify strategies to address institutional barriers to meaningful community engagement.

Engaging community members in research and healthcare improvement activities is becoming more intuitive due to the potential for making research results more relevant and healthcare more effective. Funders are increasingly requiring community input, thus creating opportunities to organize and better prepare community stakeholders. Expanded research roles and training for community members can enable them to positively impact research design, implementation, and dissemination. Expert community feedback can also influence priorities that are culturally balanced in their approach with an increased emphasis on health equity and patient-centeredness. These expanded research roles include proposal preparation, proposal review, short- and long-term consulting, community advisory board, research team member, and community principal investigator. Furthermore, healthcare regulatory and governing bodies increasingly expect patient and other community stakeholder engagement in healthcare improvement activities, such as patient advisory councils and community health needs assessments. In this interactive learning lab we examined these expanded roles for patients and other community stakeholders. Based on their life experience and firsthand knowledge of a particular community or condition, community stakeholders bring a wealth of expertise to the research enterprise. Basic training in research methods and principles, guidance on how to work with researchers, and the priorities of research institutions can help empower them in these expanded research roles. Presenters were able to build on their own experience and draw on the experiences of workshop participants as they discussed 1) how to identify and recruit community members for these roles – particularly groups traditionally underrepresented in research and underserved by healthcare institutions, 2) best practices to adequately prepare community members to serve as consultants, advisors, and team members for research and healthcare improvement activities, and 3) effective strategies to address barriers to meaningful community engagement within the institution.

## LL11 Promotores (community health workers) as partners in research: lessons learned and recommendations

### Katrina Kubicek

#### Clinical Translational Science Institute, University of Southern California, Los Angeles, CA, USA

The goal of this learning lab was to help clarify some of these questions through a presentation and roundtable dialogues. We discussed the Promotore model and the emerging trends of 1) integrating Promotores into research teams, 2) examples of what works, 3) challenges when integrating Promotores in research, and 4) lessons learned. Our work in the last five years has allowed us to learn more about the capacity of Promotores as partners in research recruitment, data collection, and consultants in the development of data collection tools and recruitment strategies. This experience has led our team to hire four Promotores as permanent staff members.

To address our national goal of “achieving health equity, eliminating disparities, and improving the health of all groups,” innovative approaches are needed to fully engage diverse communities in the conduct and dissemination of research. Engaging the community in this process can have a positive impact on decreasing this gap. Community members can serve as catalysts and “change agents” in efforts to develop, evaluate, and implement interventions designed to improve human health across a wide range of human diseases and conditions. Many have found that Promotores – individuals who have close ties to the communities they serve – can be effective partners in research when evaluating health interventions. Through their close relationships in their communities, Promotores have built trusting relationships, something invaluable for public health research and promotion. Over the last several years, our team has seen an increase in interest from researchers wanting to integrate Promotores into their work. As a result, there are many questions, uncertainties, and requests for consultations and work to determine what roles are most relevant for a Promotore or Community Health Worker.

